# The Independent Walking for Brain Health Intervention for Older Adults: Protocol for a Pilot Randomized Controlled Trial

**DOI:** 10.2196/42980

**Published:** 2023-02-13

**Authors:** Chelsea C Hays Weeks, Alison A Moore, Matthew Allison, Kevin Patrick, Mark W Bondi, Camille Nebeker, Thomas T Liu, David Wing, Michael Higgins, Sheri J Hartman, Robert A Rissman, Zvinka Z Zlatar

**Affiliations:** 1 Veterans Affairs San Diego Healthcare System San Diego, CA United States; 2 Department of Medicine University of California, San Diego La Jolla, CA United States; 3 Department of Family Medicine University of California, San Diego La Jolla, CA United States; 4 Herbert Wertheim School of Public Health and Human Longevity Science University of California, San Diego La Jolla, CA United States; 5 Department of Psychiatry University of California, San Diego La Jolla, CA United States; 6 Department of Radiology University of California, San Diego La Jolla, CA United States; 7 Department of Neurosciences University of California, San Diego La Jolla, CA United States

**Keywords:** older adults, seniors, real world, real time, digital health, feasibility, brain perfusion, cognition, adaptive intervention, exercise, clinical trial, mechanisms

## Abstract

**Background:**

Extensive research suggests that physical activity (PA) is important for brain and cognitive health and may help to delay or prevent Alzheimer's disease and related dementias. Most PA interventions designed to improve brain health in older adults have been conducted in laboratory, gym, or group settings that require extensive resources and travel to the study site or group sessions. Research is needed to develop novel interventions that leverage mobile health (mHealth) technologies to help older adults increase their engagement in PA in free-living environments, reducing participant burden and increasing generalizability of research findings. Moreover, promoting engagement in moderate-to-vigorous PA (MVPA) may be most beneficial to brain health; thus, using mHealth to help older adults increase time spent in MVPA in free-living environments may help to offset the burden of Alzheimer's disease and related dementias and improve quality of life in older age.

**Objective:**

We developed a novel PA intervention that leverages mHealth to help older adults achieve more minutes of MVPA independently. This pilot study was a 12-week randomized controlled trial to investigate the feasibility of providing just-in-time (JIT) feedback about PA intensity during free-living exercise sessions to help older adults meet current PA recommendations (150 minutes per week of MVPA).

**Methods:**

Participants were eligible if they were cognitively healthy English speakers aged between 65 and 80 years without major cardiovascular, neurologic, or mental health conditions; could ambulate independently; and undergo magnetic resonance imaging. Enrollment occurred from October 2017 to March 2020. Participants randomized to the PA condition received an individualized exercise prescription and an mHealth device that provided heart rate–based JIT feedback on PA intensity, allowing them to adjust their behavior in real time to maintain MVPA during exercise sessions. Participants assigned to the healthy aging education condition received a reading prescription consisting of healthy aging topics and completed weekly quizzes based on the materials.

**Results:**

In total, 44 participants were randomized to the intervention. A follow-up manuscript will describe the results of the intervention as well as discuss screening, recruitment, adverse events, and participants’ opinions regarding their participation in the intervention.

**Conclusions:**

The long-term goal of this intervention is to better understand how MVPA affects brain and cognitive health in the real world and extend laboratory findings to everyday life. This pilot randomized controlled trial was conducted to determine the feasibility of using JIT heart rate zone feedback to help older adults independently increase time spent in MVPA while collecting data on the plausible mechanisms of change (frontal and medial temporal cerebral blood flow and cardiorespiratory fitness) that may affect cognition (memory and executive function) to help refine a planned stage 2 behavioral trial.

**Trial Registration:**

ClinicalTrials.gov NCT03058146; https://clinicaltrials.gov/ct2/show/NCT03058146

**International Registered Report Identifier (IRRID):**

DERR1-10.2196/42980

## Introduction

### Background

It is projected that Alzheimer's disease and related dementias (ADRD) diagnoses in people aged ≥65 years will increase by 22% from 2020 to 2025 [1]. Given the lack of effective treatments for ADRD, interventions that focus on healthy lifestyle choices to prevent cognitive decline and bolster brain health are important. In this regard, the evidence suggests that the modifiable lifestyle factors most strongly associated with ADRD risk include midlife obesity, physical inactivity, and low education [2] and that approximately 21% of Alzheimer's disease cases in the United States are thought to be attributable to physical inactivity [3]. Importantly, up to 40% of dementia cases could be prevented by management of modifiable risk factors, including physical inactivity [4]. Moreover, strong evidence links physical activity (PA) with a reduction in age-related cognitive decline and dementia risk [4-6]. For example, higher PA is associated with 14% lower risk of dementia when compared with those with lower levels of PA [7], whereas, compared with sedentary individuals, even low-to-moderate PA has been associated with a 35% reduction of risk for cognitive decline [8]. Despite this, just 46% and 32.3% of individuals aged 65 to 74 years and ≥75 years, respectively, met the 2008 Physical Activity Guidelines for Americans [9], and 29.4% of people aged ≥65 years reported performing no PA other than their job in the past 30 days in 2020 [10]. The 2018 Physical Activity Guidelines for Americans issued by the US Department of Health and Human Services recommend that older adults achieve at least 150-300 minutes per week of moderate-intensity PA or 75-150 minutes per week of vigorous-intensity PA, perform muscle-strengthening activities on ≥2 days per week, do balance training, and “move more and sit less” throughout the day [11,12]. Traditionally, the cognitive and ADRD-preventing benefits of PA have been more strongly linked to changes in moderate-to-vigorous PA (MVPA) [13] that can lead to improvements in cardiorespiratory fitness [14,15]. As such, interventions that target MVPA are needed to help older adults achieve current PA guidelines and prevent ADRD in late life.

It should be noted that most interventions that investigate how changes in MVPA affect cognition and brain health have been conducted in controlled settings or under supervised conditions. These interventions are difficult to implement on a large scale and are less generalizable than those conducted in a person’s natural environment. Research is needed to develop novel interventions to help older adults increase their engagement in MVPA in free-living environments. Notably, mobile health (mHealth) technology (eg, smartwatches and heart rate trackers) can help fill this gap and extend laboratory findings to the real world by helping older adults achieve PA recommendations independently, reaching more individuals, and potentially increasing adoption and adherence [16,17].

To maximize the efficacy of mHealth interventions, there is a need for these technologies to offer adaptive and just-in-time (JIT) feedback to help individuals change their behavior in real time [18,19], as well as provide social support to help promote behavior change [20]. For example, leveraging heart rate tracking to provide JIT support about PA intensity in real time during an exercise session can cue individuals to change their walking pace accordingly and spend more time in MVPA during an exercise bout. This JIT support can also help with self-monitoring, which is an important component of successful mHealth PA interventions [20]. Although mHealth use may at times be challenging for some older adults, there is low-to-moderate evidence that mHealth interventions may help to increase PA in older adults in the short term [20], and research supports the effectiveness of mHealth approaches in those aged ≥50 years [21]. Similarly, tracking with mHealth devices has been shown to enhance the positive effect of PA levels on satisfaction with physical fitness [22], and older adults find that mHealth devices that provide JIT feedback can help them to walk faster during free-living exercise sessions [23]. Overall, research suggests that mHealth can facilitate behavior change in older age [24-26].

### Objectives

The Independent Walking for Brain Health study was a 12-week small pilot randomized controlled trial (RCT) conducted to determine the feasibility of using JIT heart rate–based feedback to help older adults independently meet PA recommendations of 150 minutes of MVPA per week [12,27], while collecting data on the plausible mechanisms of change (frontal and medial temporal cerebral blood flow [CBF] and cardiorespiratory fitness) that may affect cognition (memory and executive function) to help refine a planned stage 2 behavioral trial. We hypothesized that the heart rate–based JIT feedback would help older adults to increase the time they spent in MVPA during free-living exercise sessions [23]. The long-term goal of this intervention is to better understand how MVPA affects brain and cognitive health in the real world and extend laboratory findings to everyday life. Participants randomized to the PA condition (PAC) were provided with personalized walking prescriptions, weekly performance summary emails, goal setting, and regular telephone calls from study staff to review achievements and revise goals if needed. JIT mHealth strategies included immediate haptic and visual alerts when the participants deviated from their individualized heart rate target zone during an exercise session. Participants in the healthy aging education condition (HAEC) received a reading prescription and answered quizzes based on the materials. We collected accelerometer data, cardiorespiratory fitness data, brain imaging data, and cognitive performance data to evaluate the feasibility of the study procedures, as well as daily-level heart rate data from the mHealth device that participants used to record their exercise sessions. This protocol paper describes the Independent Walking for Brain Health pilot RCT.

## Methods

### Study Procedures

**Figure 1:**
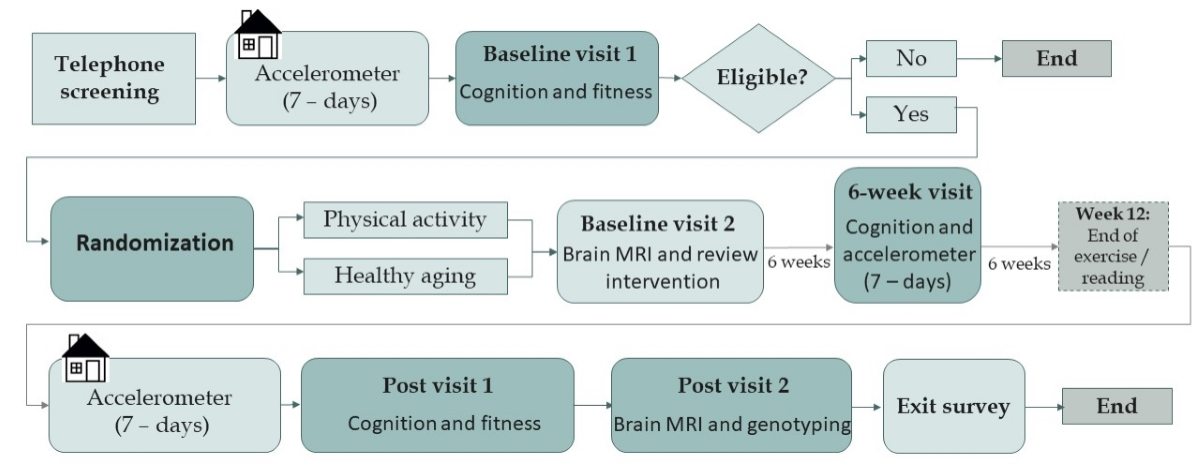
Study flow. MRI: magnetic resonance imaging.

The Independent Walking for Brain Health study was a 12-week free-living pilot RCT to determine the feasibility of using JIT heart rate–based feedback to help older adults independently meet PA recommendations of 150 minutes of MVPA per week. Participants who met the inclusion criteria (described in the next section) were randomly assigned to the PAC or HAEC and were provided with either a walking prescription or a reading prescription, respectively. All aspects of the intervention took place remotely, aside from the measurement visits, which were conducted at the University of California San Diego at baseline, 6 weeks, and 12 weeks. More details about each component of the intervention and the measures collected are described in the sections that follow. Table 1 shows the assessments collected and the time interval in which they were administered. Figure 1 depicts the study flow and procedures.

**Table 1:** Measures administered and schedule of administration.

Measure		Phone screen	Baseline	6 weeks	12 weeks	Continuous
**Demographic and medical history**						
	Basic demographics	✓				
	Self-reported physical activity	✓				
	Eligibility criteria	✓	✓			
	Physician’s clearance	✓				
	Framingham Stroke Risk Profile		✓			
	Blood pressure, heart rate, weight, and height		✓		✓	
	mHealth^a^ heart rate tracking					✓
**Cognition**						
	Telephone interview for cognitive status	✓				
	Mattis Dementia Rating Scale		✓			
	Rey Auditory Verbal Learning Test		✓	✓	✓	
	Trail-Making Test		✓	✓	✓	
	Stroop Color Word Interference Test		✓	✓	✓	
	Verbal Fluency		✓	✓	✓	
	WMS-R^b^ Logical Memory I and II		✓	✓	✓	
	NIH^c^ Toolbox Cognition Battery		✓	✓	✓	
Accelerometer measurement (7 days)			✓	✓	✓	
Submaximal GXT^d^ treadmill test			✓		✓	
Brain magnetic resonance imaging			✓		✓	
Apolipoprotein E genotype					✓	
**Questionnaires**						
	Geriatric Depression Scale		✓	✓	✓	
	Sedentary Behavior Questionnaire		✓	✓	✓	
	Everyday Cognition Questionnaire		✓	✓	✓	
	Subjective Cognitive Decline Questionnaire		✓	✓	✓	
	PROMIS^e^ Anxiety		✓	✓	✓	
	PROMIS Depression		✓	✓	✓	
	PROMIS Social Sat DSA^f^		✓	✓	✓	
Exit survey					✓	

^a^mHealth: mobile health.

^b^WMS-R: Wechsler Memory Scale, revised version.

^c^NIH: National Institutes of Health.

^d^GXT: graded exercise testing.

^e^PROMIS: Patient-Reported Outcomes Measurement Information System.

^f^Social Sat DSA: Satisfaction with Participation in Discretionary Social Activities.

### Participant Recruitment and Inclusion Criteria

Participants were recruited from ongoing studies at the Wellness Initiative for Senior Enrichment laboratory at the University of California San Diego and the Shiley-Marcos Alzheimer’s Disease Research Center, as well as from ResearchMatch [28], flyers, community engagement talks (ie, talks at retirement communities, senior centers, libraries, and health fairs) and by word of mouth. All participants were enrolled between October 2017 and March 2020, at which time study recruitment was terminated because of the COVID-19 pandemic.

All potential participants were prescreened via telephone to determine basic eligibility, which included (1) age between 65 and 80 years, (2) able to obtain a signed physician’s clearance for participating in PA, (3) English speaking, (4) not meeting the current PA recommendations of 150 minutes of MVPA per week (via self-report), (5) not reporting contraindications for magnetic resonance imaging (MRI), and (6) able to ambulate independently. Participants were excluded if they had (1) objective evidence of cognitive impairment during the telephone screen (scores ≤34 on the modified Telephone Interview for Cognitive Status [29]) or during baseline (first in-person visit) neuropsychological testing (performance >1 SD below demographically adjusted norms on at least 2 measures within the same cognitive domain [30]); (2) history of head injury involving loss of consciousness within the past 6 months; (3) major neurological disorders (eg, dementia, multiple sclerosis, or epilepsy); (4) chronic major psychiatric disorders (eg, schizophrenia or bipolar disorder); (5) history of major vascular events (eg, myocardial infarction or stroke); (6) history of diabetes; (7) history of falls in the last year resulting in hospitalization; and (8) unstable or severe medical problems (eg, uncontrolled heart failure or hypertension, pulmonary disease with hypoxia or hypercapnia, significant liver problems, or renal failure), treatment of cancer (other than nonmelanoma skin cancer) in the past 2 years, or were HIV positive.

### Ethics Approval

All study procedures were reviewed and approved by the institutional review board of the University of California, San Diego (IRB #151278), and abided by the declaration of Helsinki. Written informed consent was gathered from all participants after a thorough discussion of the risks and benefits of participating in the program. All data were deidentified and maintained in a Health Insurance Portability and Accountability Act–compliant server (Research Electronic Data Capture [REDCap]; Vanderbilt University). All participants were compensated up to US $200 for participating in the in-person study visits.

### Study Design

The Independent Walking for Brain Health pilot study was a 12-week RCT designed to test the feasibility of using an mHealth device to assist older adults independently achieve recommended PA guidelines (≥150 minutes per week of MVPA) in their free-living environments. Only the participants randomized to the PAC received an individualized exercise prescription and a commercially available mHealth device that provided heart rate–based JIT feedback regarding PA intensity during exercise sessions. Those assigned to the HAEC received a reading prescription about healthy aging topics and completed weekly quizzes based on the materials. More details are provided in the following sections.

### Randomization Procedures

We planned to recruit 60 older adults to be randomized to the HAEC (n=30, 50%) or PAC (n=30, 50%). Because of the COVID-19 pandemic, only 44 participants were randomized before study termination, with 23 (52%) in the HAEC and 21 (48%) in the PAC. Stratified blocked randomization was used to ensure a similar balance of age (block 0: 65-72 years and block 1: 73-80 years) and sex (block 0: male and block 1: female) between the intervention conditions (block 0: HAEC and block 1: PAC). The randomization table was generated by an independent statistician and uploaded to the REDCap database. Participants were randomized to the PAC or HAEC after completing their first baseline visit to ensure that they met our cognitive testing inclusion criteria and were able to safely perform the cardiorespiratory fitness test. Participants who did not meet our cognitive testing inclusion criteria or did not pass the treadmill test were withdrawn from the study before randomization (Figure 1). Randomization was performed by the REDCap database using the randomization schedule randomly created by the statistician. Given the pilot nature of the study, the principal investigator (PI) and measurement staff were not blinded to the participants’ assigned condition.

### Intervention Arms

#### PAC Description

The main goal of the PAC was to use the mHealth device as a *coach* to provide JIT support to prompt real-time changes in PA intensity to maintain MVPA. Participants received individualized walking prescriptions based on heart rate targets equivalent to MVPA obtained from submaximal graded exercise testing (GXT) at baseline. As such, each participant’s heart rate target for the MVPA prescription was different based on their GXT performance (described in the Measures section). The individualized exercise prescriptions were built to gradually increase the intensity of PA and the number of minutes spent in MVPA to 150 per week. Refer to Table 2 for an example of the exercise prescription. Although the prescriptions were based on walking, participants were encouraged to perform any purposeful activity that increased their heart rate above their individualized minimum target equivalent to MVPA. Participants who did not meet the weekly number of MVPA minutes in the prescription during a given week were encouraged to increase MVPA by 10 minutes the following week. The exercise prescription also included prompts for performing strength and flexibility exercises weekly (ankle circles, toe lifts, partial stands, knee flexion and extension, and wrist, shoulder, calf, and shin stretches) accompanied by pictures and descriptions to guide participants on how to perform these exercises safely at home. As our focus was on increasing MVPA, we did not track progress or engagement in the strength and flexibility exercises.

**Table 2:** Exercise prescription. Prescribed number of minutes per week within each intensity level.

Intervention week	Slow minutes	Moderate minutes	Fast minutes	MVPA^a^ (moderate+fast) minutes	All active minutes per week
1	50	20	0	20	70
2	60	20	10	30	90
3	30	40	15	55	85
4	0	70	15	85	85
5	0	90	30	120	120
6	30	60	35	95	125
7	0	100	35	135	135
8	30	100	25	125	155
9	0	110	35	145	145
10	0	115	35	150	150
11	0	125	35	160	160
12	0	125	35	160	160

^a^MVPA: moderate-to-vigorous physical activity.

**Figure 2:**
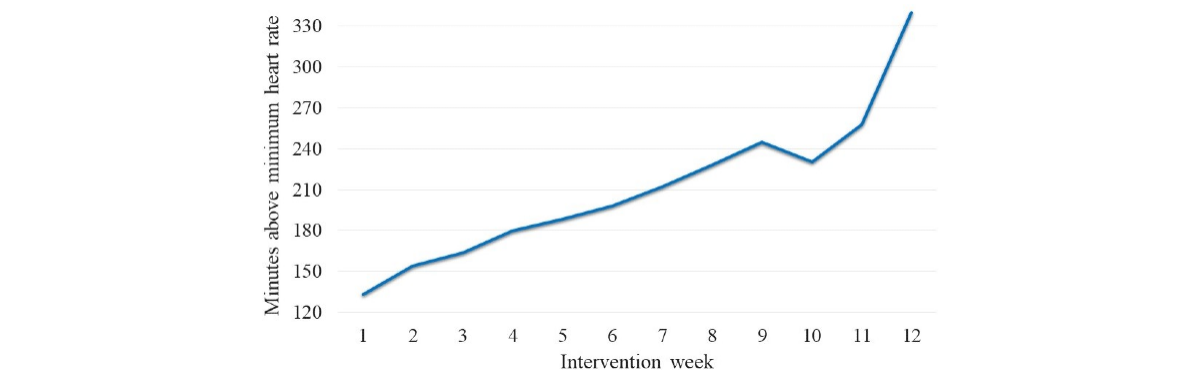
Example progress graph emailed weekly to participants in the physical activity condition to review moderate-to-vigorous physical activity goals and set new goals for the following week. The y-axis shows the number of minutes the participant spent above their minimum moderate-to-vigorous physical activity heart rate target for each week based on tracking from the mobile health device. The x-axis depicts the treatment week.

During the randomization visit, participants in the PAC received their individualized walking prescriptions, the mHealth device, and a folder containing readings about PA, safety, and troubleshooting the mHealth device, as well as important contact information and a detailed schedule of upcoming telephone calls and in-person appointments. Participants were also coached on how to use the mHealth device and respond to the JIT feedback during a walking session with study staff. Participants received a weekly personalized email summarizing the number of minutes spent in MVPA for that week (Figure 2), which also delineated their MVPA goal for the following week. Weekly telephone calls from study staff (ZZZ, the PI, who is a licensed clinical neuropsychologist) lasted 10 to 15 minutes and were conducted during the first month, followed by biweekly calls during months 2 and 3. The telephone calls focused on reviewing the minutes of MVPA achieved, identifying challenges to engaging in exercise, revising goals as needed, and checking on each participant’s safety.

At home, the mHealth device tracked participant heart rate during each exercise session, providing JIT haptic and visual feedback in real time (blue=below zone, green=in zone, and red=above zone) when they deviated from their prescribed heart rate target zone. This allowed them to correct their walking pace or exercise intensity in real time. Participants were encouraged to exercise above their minimum heart rate equivalent to MVPA, but they were not discouraged from going above their target zone if they felt it was safe to do so. Minute-by-minute heart rate data gathered from the mHealth device was saved to the device and later synced with the corresponding app and emailed to the study team on a regular basis. This helped with accountability and tracking by the study team to provide weekly feedback about progress via emails and telephone calls. Participants’ perspectives about using the mHealth device in this study have been described elsewhere [23].

#### HAEC Description

Participants assigned to the HAEC received a reading prescription that equated the time spent reading study materials to the time those in the PAC spent engaging in PA (approximately 2.5 hours per week). The reading prescription consisted of 50 short or bulleted chapters to be completed over 12 weeks on topics that included retirement, quality of life, talking to your physician, bladder health, pain, preventing falls, legal and financial planning, heart health, nutrition, stress management, and humor. There were 4 to 5 chapters (the number of chapters varied based on chapter length) emailed to participants every week, which they could read at their own pace. Participants received a weekly quiz via REDCap that required them to answer 3 to 5 questions about each chapter to measure adherence to the reading prescription. Participants received weekly telephone calls from study staff during the first month, followed by biweekly calls during months 2 and 3. The purpose of the telephone calls, which lasted 5 to 10 minutes, was for the participants to receive equal amounts of attention from study staff between the conditions as well as for the study staff to answer any questions related to the materials and check on the participant’s health status. All the chapters were prepared by the investigative team using materials freely available from the National Institute on Aging and Alzheimer’s Association websites among other reputable sources. Textbox 1 lists the reading prescription topics assigned per month.

**Reading prescription topics assigned to participants in the healthy aging education condition.**

**Month and topics**

1. Retirement, driving, immunizations, health care, prostate and bladder health, pain, memory, eye health, medications, heart health, disability, taste, smell, frontotemporal disorders, quality of life, and talking to your physician
2. Mental health, dementia, preventing falls, antiaging tips, leukemia, disease prevention, hearing loss, arthritis and osteoporosis, legal and financial planning, mild cognitive impairment, Alzheimer's disease, lifestyle and longevity, sexual health, high blood pressure, Alzheimer's disease caregiving, and humor
3. Heart disease, stress management, gum disease, alternative medicine, headaches and migraines, diabetes, myocardial infarction, nutrition, preventive medicine, skin care and aging, sleep and aging, social media, stroke, constipation, hydration, food for thought, and happiness in the home and the community

### Measures

#### Cognitive Testing

In a quiet room with a trained study staff member, participants completed the Mattis Dementia Rating Scale [31]; the National Institutes of Health (NIH) Toolbox Cognition Battery [32] (including the optional Oral Symbol Digit Test); the Rey Auditory Verbal Learning Test (RAVLT) [33]; the Golden version of the Stroop Color Word Interference Test [34]; the Wechsler Memory Scale-revised Logical Memory I and II [35]; Trail-Making Test parts A and B [36]; and verbal fluency tests (FAS Test [assessment of phonemic fluency by requesting an individual to orally produce as many words as possible that begin with the letters F, A, and S within 1 minute] and animal naming) [37]. As executive and memory functions are most responsive to exercise in intervention trials with older adults [14], we created executive and memory composite scores by converting raw scores into *z* scores based on the entire sample, and then averaging across *z* scores for the following tests:

- Executive composite scores: Trail-Making Test part B minus Trail-Making Test part A (scores were reversed before averaging to reflect higher scores=better performance), Stroop Color Word Trial, and verbal fluency FAS Test
- Memory composite scores: Wechsler Memory Scale-revised Logical Memory I and II; RAVLT trials 1 to 5, trial 6 (short-delay free recall), and RAVLT delayed recall

We picked these measures because they have previously been reported in the PA intervention literature and have been shown to change as a function of exercise [14].

#### PA Measurement

PA was measured using triaxial accelerometers (GT3X; ActiGraph, LLC) for 7 consecutive days [38,39]. Participants were instructed to wear the accelerometer on a belt on their hip during waking hours for a minimum of 12 hours per day. They were also instructed not to change their regular activities. To ensure compliance, all participants received 2 telephone calls from study staff (on days 2 and 5 of the monitoring period). Participant data were considered valid only if they had accumulated a minimum of 600 minutes of accelerometer wear per day and a minimum of 3000 total minutes of wear spread across at least 4 valid days. Data were processed using ActiLife software (version 6.0; ActiGraph, LLC). Using the low-frequency extension option thought to be most appropriate for older adults [40], data were aggregated to 60-second epochs so that published cut points could be applied. Consistent with standard practice, sedentary time was defined as time spent at <100 counts per minute and MVPA as ≥1952 counts per minute [41]. Minutes within each intensity level were then averaged across days worn, reflecting the average time in minutes per day spent at each intensity level.

#### Cardiorespiratory Fitness Measurement and Heart Rate Target Zones for the Exercise Prescription

Cardiorespiratory fitness was measured via submaximal GXT protocol at baseline and at 12 weeks. During the treadmill test, heart rate was plotted against the workload and its corresponding estimated VO_2_ [42], and VO_2_ max was estimated plotting the slope of the line to estimated maximal heart rate (calculated using the formula 220–age). A 1-minute warm-up at 2 mph (3.2 km/h) was used to familiarize participants with the treadmill and give them time to adjust to walking without holding the handrails. After the warm-up, the speed was increased to 2.5 mph (4 km/h) at 0% grade, equivalent to 2.9 metabolic equivalents (METs), which is just below the threshold for moderate-intensity exercise. This stage lasted 3 minutes to ensure that steady state was achieved. The speed was then increased to 3 mph (4.8 km/h) at 0% grade, equivalent to 3.3 METs, which is just above moderate-intensity exercise. This stage lasted 5 minutes. One-minute stages with grade increments of 1.5% (0.62 METs) per stage continued until the participant’s heart rate reached volitional fatigue. Heart rate was monitored continuously using a Polar heart rate monitor (Polar FT4 or similar; Polar Electro) throughout the test and for at least 5 minutes of recovery. Blood pressure was recorded for the first 3 stages of exercise and every minute during recovery, at which time participants continued walking at a very slow pace (1 mph; 1.6 km/h) for 2 minutes, followed by 3 minutes of sitting recovery with light foot tapping to prevent venous pooling.

The heart rates measured during the final 10 seconds of minutes 7 and 9 of the GXT were used to determine heart rate target zones for the exercise prescription.

These heart rate measurement times occurred during stage 3 (3 mph; 4.8 km/h) at which participants are estimated to be working at a level equivalent to 3.3 METS, a threshold slightly higher than the minimum level to be considered *moderate* activity. Minutes 7 and 9 of the total protocol were chosen because of the likelihood that participants would have reached *steady state* after ≥3 minutes at that workload. Steady state is the point at which the heart’s work is equal to the workload and is unlikely to change with increased exercise duration. For exercise prescription purposes, the 2 heart rate values gathered during minutes 7 and 9 of the total protocol were averaged if the difference was ≤3 beats per minute, and the greater of the 2 heart rates was used if the difference was >3 beats per minute. Participants were asked to refrain from consuming caffeine or other stimulants on the day of their test.

For this pilot RCT, we were interested in examining the plausible mechanisms by which changes in MVPA may affect cognition, including changes in cardiorespiratory fitness. We defined changes in cardiorespiratory fitness as the total time to 85% of estimated maximal heart rate (with an exception made for participants taking β-blockers, as described later in this paragraph) and changes in rate-pressure product (RPP), which is the product of heart rate and systolic blood pressure divided by 100 measured toward the end of each of the first 3 stages of exercise. RPP is an indirect assessment of cardiac workload. A decrease in RPP for a given amount of aerobic work often signifies an improvement in cardiac function [43]. Individuals taking β-blockers were asked to exercise to a workload corresponding to 80% of their age-predicted maximal heart rate. This precaution accounts for the approximately 5% reduction in heart rate observed with taking β-blockers and allowed the research team to provide scaled exercise prescriptions.

#### Brain MRI Measurement and Processing

MRI data were acquired on a GE Discovery MR750 3.0T (General Electric Company) whole body system with a body transmit coil and an 8-channel receive-only head coil at the University of California, San Diego Center for Functional MRI. The structural brain sequence consisted of a high-resolution T1-weighted fast spoiled gradient echo (3DFSPGR) scan for anatomy and registration purposes: 172 1-mm contiguous sagittal slices, field of view=25 cm, repetition time (TR)=8 ms, echo time (TE)=3.1 ms, flip angle=12°, inversion time=600 ms, 256 × 192 matrix, bandwidth=31.25 kHz, frequency direction=S-I, NEX=1, and scan time=8:13 minutes. CBF was quantified with a 2D pseudocontinuous arterial spin labeling (ASL) MRI sequence: TR=4500 ms, TE=3.2 ms, field of view=24 cm, labeling duration=1800 ms, postlabeling delay=2000 ms, with a single-shot spiral acquisition and a total scan time of 4:30 minutes plus a 40.5-second calibration scan. The calibration scan was acquired immediately after the ASL scan using a spiral readout with TR=4.5 seconds and TE=3.2 ms with 8 dummy radiofrequency pulses (amplitude set to 0) to generate a 36-second delay followed by a 90-degree radiofrequency pulse in the last repetition interval to generate proton density–weighted contrast. Field map scans were collected for offline field map correction to minimize signal bunching and dropouts in the frontal or medial temporal lobes.

T1-weighted anatomical images were processed using FreeSurfer software (version 6.0). Briefly, images underwent skull stripping, B1 bias field correction, segmentation of gray matter and white matter, reconstruction of cortical surface models, and parcellation and labeling of regions on the cortical surface, as well as segmentation and labeling of subcortical structures [44]. FreeSurfer was also used to generate intracranial volume. ASL data were processed using the Cerebral Blood Flow Biomedical Informatics Research Network (CBFBIRN) [45] pipeline established by the University of California, San Diego Center for Functional MRI. CBFBIRN uses a combination of custom MATLAB (The MathWorks, Inc) [46] routines and various Analysis of Functional NeuroImages [47] and FMRIB Software Library [48] functions to quantify CBF and adjust for partial volume effects. MATLAB was used to form a mean ASL image from the average difference of the control and tag images. Voxelwise CBF calibration was performed using the proton-density image to convert the ASL difference signal into physiological units (mL/100 g/min). Slice-timing delays were also accounted for, making the postlabeling delay slice specific. Skull stripping of the high-resolution T1-weighted image was performed using the Analysis of Functional NeuroImages 3dSkullStrip function. Tissue segmentation was performed using the FMRIB Software Library Automated Segmentation Tool algorithm to define cerebrospinal fluid, gray matter, and white matter regions. The high-resolution T1-weighted image and partial volume segmentations were registered to ASL space. A linear regression method was used to correct for partial volume effects and ensure that CBF values were not influenced by decreased perfusion in the white matter or increased volume of cerebrospinal fluid [49], with a 5 × 5 regression kernel to obtain corrected gray matter CBF measurements. Each participant’s partial volume–corrected quantified CBF map (in units of mL/100 g tissue/min) was downloaded to a local server. Voxels with negative intensities were replaced with 0. FreeSurfer was used to generate a priori anatomical regions of interest for the CBF data. Briefly, for each participant, the FreeSurfer-formatted T1-weighted brain volume was registered to the ASL CBF-aligned T1-weighted anatomical image (the latter was derived as part of the CBFBIRN pipeline). The resulting coregistration matrix was used to align the FreeSurfer aparc+aseg segmentation volume to the ASL CBF-aligned T1-weighted image. The CBF-aligned FreeSurfer volumes were visually inspected to ensure proper alignment and were then downsampled to the resolution of the CBF ASL image. Mean CBF was then extracted for FreeSurfer regions of interest. For this pilot RCT, we were interested in examining the plausible mechanisms by which changes in MVPA may affect cognition, including changes in total gray matter CBF, frontal lobe CBF (mean of superior frontal, rostral and caudal middle frontal, pars opercularis, pars triangularis, pars orbitalis, lateral and medial orbitofrontal, precentral, paracentral, and frontal pole CBF), and medial temporal lobe CBF (mean of entorhinal, parahippocampal, and hippocampal CBF).

#### Apolipoprotein E Measurement

Saliva samples were collected using the Isohelix SK-2S DNA buccal swab kit (Cell Projects Ltd) to provide information regarding genetic risk for Alzheimer's disease. Genotype for apolipoprotein E was determined by real-time analysis of single nucleotide polymorphism using TaqMan technology (Life Technologies). The real-time analysis was performed using validated TaqMan assays on a CFX96 Touch Real-Time Polymerase Chain Reaction Detection System (Bio-Rad Laboratories, Inc) according to the manufacturer’s instructions. Analysis was performed using Bio-Rad CFX Manager software.

### Monitoring Adverse Events and Data Safety and Monitoring Plan

All adverse events were carefully monitored via telephone contact with participants throughout the trial and were reported to the institutional review board of the University of California, San Diego, and the National Institute on Aging program official according to NIH guidelines. A data safety and monitoring plan was developed for this study focusing on close monitoring by the PI (ZZZ) and research staff, in conjunction with oversight from a safety officer (AAM, a practicing geriatrician). A future publication of the pilot trial’s results will report all adverse events.

### Data Management and Quality Control

All study staff completed NIH-required human research participants training as well as thorough training by the study PI (ZZZ) on how to perform telephone screenings, administer and score the cognitive tests, and interact with participants, as well as on ethical aspects of clinical trials research and safe data monitoring practices. All cognitive testing data were double scored by 2 different staff members and entered into the REDCap database, which was also double-checked for accuracy. Accelerometer and cardiorespiratory fitness data were collected by professionals at the University of California, San Diego Exercise and Physical Activity Resource Center, which specializes in collecting these measurements with highly trained exercise physiologists. All brain imaging data were collected and analyzed by highly trained study personnel, including the PI. Study protocols were strictly followed to ensure scientific rigor and allow for reproducibility.

## Results

Enrollment for this study began in October 2017 and was terminated early in March 2020 as a result of the COVID-19 pandemic. In total, 44 participants were randomized to the intervention, with 21 (48%) in the PAC and 23 (52%) in the HAEC. In keeping with the CONSORT (Consolidated Standards of Reporting Trials) guidelines for pilot and feasibility trials [50,51], the results from this pilot intervention will focus on discussing the feasibility of the JIT mHealth alerts to increase MVPA, presenting estimates of variability in response to the outcome and plausible mechanisms, presenting recruitment and retention numbers and challenges, discussing adherence by study condition, discussing adverse events, discussing the ability to implement the intervention or lessons learned, and summarizing the participants’ opinions regarding the intervention. The results, which will be reported in a separate manuscript, will inform the optimization of a planned stage 2 behavioral intervention trial to help maintain cognitive function through increases in MVPA using mHealth.

## Discussion

### Overview

This protocol paper describes the Independent Walking for Brain Health pilot RCT. The study was an innovative mHealth trial to determine the feasibility of using heart rate–based JIT feedback to help older adults maintain MVPA during free-living exercise sessions. This pilot trial also explored the mechanisms, namely changes in CBF and cardiorespiratory fitness, by which increasing MVPA via JIT alerts may influence cognitive function. Between October 2017 and March 2020, a total of 44 participants were randomized to the intervention, with 21 (48%) in the PAC and 23 (52%) in the HAEC. A follow-up manuscript will describe the results of the pilot intervention.

The literature shows that MVPA, performed under supervised conditions (in the laboratory, gym, or supervised by a trainer) is important for maintaining brain health [52-55] and can boost cognitive performance in older age [56-59]. However, there is a lack of interventions that leverage mHealth to help older adults increase their engagement in MVPA independently, which would generalize study findings to the real world and remove some of the barriers to performing PA (eg, travel to the gym and monetary constraints). This pilot RCT is the first step in the refinement of a future stage 2 behavioral trial to help older adults achieve and maintain MVPA independently to help support their cognition and brain health. The findings from this pilot RCT will help inform the larger trial and refine the plausible mechanisms of change, as well as determine whether JIT heart rate–based feedback can help older adults to increase time spent in MVPA independently.

### Limitations

The pilot study includes the following limitations:

1. A short intervention time (12 weeks), which is not sufficient to observe changes in cognitive function [60]. Future mHealth interventions should be of longer duration.
2. The mHealth device used in this study was not specifically designed for older adults. Future trials should use devices that have been tested with older adults to increase the likelihood of adherence to, and acceptability of, the intervention.
3. Importantly, this trial focused on promoting only MVPA because evidence suggests that MVPA is needed to maintain brain health and perhaps prevent ADRD [13]. That said, we did encourage participants to perform stretching and toning exercises twice per week (and provided them with related materials) to follow the current PA guidelines.
4. Most recently, dementia prevention interventions have followed a multimodal approach, targeting several lifestyle factors such as diet, sleep, and cognitive training along with PA [61-67]. This study is limited in targeting only MVPA as a tool to promote brain health; however, data gathered from this study will be useful to determine whether using JIT heart rate–based feedback via mHealth successfully increases MVPA in free-living environments, which could be implemented in future multimodal interventions.

### Conclusions

In conclusion, the findings from this pilot RCT can inform future mHealth interventions that target MVPA to promote brain and cognitive health in older age and will help us to determine the utility of using JIT heart rate–based feedback to help maintain MVPA in free-living environments. Although more research is needed to determine the efficacy of JIT interventions to improve PA in older age, these interventions have the potential to be a powerful tool to help older adults lead healthier lifestyles [18,19,68]. Healthy lifestyles that include higher levels of MVPA are promising to ameliorate the burden of ADRD on the health care system and patients and their families, as well as improve the quality of life of older adults [4,63,69].

## Abbreviations

ADRD: Alzheimer's disease and related dementias
ASL: arterial spin labeling
CBF: cerebral blood flow
CBFBIRN: Cerebral Blood Flow Biomedical Informatics Research Network
CONSORT: Consolidated Standards of Reporting Trials
FAS Test: assessment of phonemic fluency by requesting an individual to orally produce as many words as possible that begin with the letters F, A, and S within 1 minute
GXT: graded exercise testing
HAEC: healthy aging education condition
JIT: just-in-time
MET: metabolic equivalent
mHealth: mobile health
MRI: magnetic resonance imaging
MVPA: moderate-to-vigorous physical activity
NIH: National Institutes of Health
PA: physical activity
PAC: physical activity condition
PI: principal investigator
RAVLT: Rey Auditory Verbal Learning Test
RCT: randomized controlled trial
REDCap: Research Electronic Data Capture
RPP: rate-pressure product
TE: echo time
TR: repetition time
